# Barriers to chronic Hepatitis B treatment and care in Ghana: A qualitative study with people with Hepatitis B and healthcare providers

**DOI:** 10.1371/journal.pone.0225830

**Published:** 2019-12-03

**Authors:** Charles Ampong Adjei, Sarah E. Stutterheim, Florence Naab, Robert A. C. Ruiter

**Affiliations:** 1 Department of Work and Social Psychology Maastricht University, Maastricht, The Netherlands; 2 School of Nursing and Midwifery University of Ghana, Accra, Ghana; The MetroHealth System and Case Western Reserve University, UNITED STATES

## Abstract

**Background:**

Hepatitis B viral (HBV) infection remains an important public health concern particularly in Africa. Between 1990 and 2013, Hepatitis B mortality increased by 63%. In recent times, effective antiviral agents against HBV such as Nucleos(t)ide analogs (NAs) are available. These drugs are capable of suppressing HBV replication, preventing progression of chronic Hepatitis B to cirrhosis, and reducing the risk of hepatocellular carcinoma and liver-related death. Notwithstanding, these treatments are underused despite their effectiveness in managing Hepatitis B. This study sought to explore barriers to treatment and care for people with Hepatitis B (PWHB) in Ghana, paying particular attention to beliefs about aetiology that can act as a barrier to care for PWHB.

**Methods:**

We used an exploratory qualitative design with a purposive sampling technique. Face-to-face interviews were conducted for 18 persons with Hepatitis B (PWHB) and 15 healthcare providers (HCP; physicians, nurses, and midwives). In addition, four focus group discussions (FGD) with a composition of eight HCPs in each group were done. Participants were recruited from one tertiary and one regional hospital in Ghana. Data were processed using QSR Nvivo version 10.0 and analysed using the procedure of inductive thematic analysis. Participants were recruited from one tertiary and one regional hospital in Ghana.

**Results:**

Three main cultural beliefs regarding the aetiology of chronic Hepatitis B that act as barriers to care and treatment were identified. These were: (1) the belief that chronic Hepatitis B is a punishment from the gods to those who touch dead bodies without permission from their landlords, (2) the belief that bewitchment contributes to chronic Hepatitis B, and (3) the belief that chronic Hepatitis B is caused by spiritual poison. Furthermore, individual level barriers were identified. These were the absence of chronic Hepatitis B signs and symptoms, perceived efficacy of traditional herbal medicine, and PWHB’s perception that formal care does not meet their expectations. Health system-related barriers included high cost of hospital-based care and inadequate Hepatitis B education for patients from HCPs.

**Conclusion:**

Given that high cost of hospital based care was considered an important barrier to engagement in care for PWHB, we recommend including the required Hepatitis B laboratory investigations such as viral load, and the recommended treatment in the National Health Insurance Scheme (NHIS). Also, we recommend increasing health care providers and PWHB Hepatitis B knowledge and capacity in a culturally sensitive fashion, discuss with patients (1) myths about aetiology and the lack of efficacy of traditional herbal medicines, and (2) patients’ expectations of care and the need to monitor even in the absence of symptoms.

## Introduction

Hepatitis B viral (HBV) infection poses a significant challenge because of its worldwide distribution and potential complications [1, 2]. Between 1990 and 2013, Hepatitis B mortality increased by 63% (0.89 million to 1.45 million), with a higher proportion occurring in Africa [3]. The majority of these deaths resulted from complications associated with HBV including cirrhosis, hepatocellular carcinoma, and liver failure [1, 4].

The enormous economic burden associated with chronic Hepatitis B [5–8] underscores the importance of early diagnosis and treatment. The American Association for the Study of Liver Disease (AASLD) practice guidelines states that people with Hepatitis B (PWHB) with elevated viral load, elevated serumalanine aminotransferase (ALT) levels, and evidence of liver fibrosis are eligible for HBV treatment [4]. In recent times, effective antiviral agents against HBV such as Nucleos(t)ide analogs (NAs) are available [1, 4]. The NAs suppress HBV replication, prevent progression of chronic Hepatitis B to cirrhosis, and reduce the risk of hepatocellular carcinoma and liver-related death [9–12]. However, despite the availability of successful treatment for HBV, in 2015, only 8% of PWHB were on treatment worldwide [1].

Although early engagement in care has shown to improve health outcomes, reduce premature deaths, and prevent complications [13], several barriers to PWHB engagement in care exist in various locales [14–17]. For example, Nwokediuko [16] noted that high costs of laboratory tests, a lack of available drugs, and lack of management guidelines are some of the challenges affecting the fight against Hepatitis B in low-income countries. In addition, fear of stigmatisation, limited infrastructure, poor resources, poor knowledge about HBV treatment, and long waiting times in hospitals have been reported as barriers to care in countries such as Australia and Burkina Faso [14–15]. Preference for herbal medicine by PWHB is also considered an important barrier to care engagement in areas such as Burkina Faso and Singapore [14, 17]. In these locales, the use of herbal medicine is widespread with some HCPs acting as referral agents to traditional herbal practitioners because they believe that traditional medicines are more effective, and less costly [14].

Generally, the behaviour of people to engage in care and treatment is dependent on several factors including the socio-cultural environment [18, 19]. The theory of explanatory model of health and illness recognises different beliefs and notions held by people regarding disease and its treatment [20, 21]. Five main dimensions of disease episodes are highlighted by Kleinman’s explanatory model: aetiology, time and mode of onset of symptoms, pathophysiology, course of sickness, and treatment [21]. In the African context, disease causation extends beyond the biopsychosocial influence to include the role of evil spirits and ancestral curses [22]. Bewitchment, spell-casting, invocation of river deities, and disregard for societal taboos are some of the beliefs associated with disease causation in Ghana [22]. With Hepatitis B in particular, previous studies in Ghana have established the perceived role that spiritual poisons, curses, and bewitchment play in its aetiology [23, 24]. Consequently, the beliefs as outlined have implications on the type of treatment used by the affected persons. According to White [22], in an event, where traditional healers establish a spiritual link to aetiology of a disease, talisman, charm, spiritual bath, and amulets are given to the person to drive away the evil spirits [22]. In some instance, domestic animals such as cats and dogs are slaughtered and buried alive at night to save the soul of the diseased person [22]. In the Christian religious circles, affected persons are often kept in prayer camps in the midst of fasting and restrained by chains as part of disease treatment process [25].

In Ghana, prevalence of chronic Hepatitis B is 12.3% [26] and HBV was, in a sample of 70 patients with cirrhosis, present in 42.9% of those patients [27]. Engagement in care for PWHB is crucial if we are to reach the Sustainable Development Goal (SDG) target of reducing Hepatitis B mortality by 65% before 2030 [28]. To our knowledge, there are two other studies conducted in Ghana that investigated the perception of pregnant women [24] and the general public [23] regarding HBV. However, none of the aforemntioned studies explored the influences of the beliefs associated with HBV on treatment. This present study thus aimed to explore barriers to treatment and care for PWHB in Ghana, paying particular attention to beliefs about aetiology that can act as a barrier to treatment, choice of treatment, and adherence to treatment for PWHB.

## Methods

### Study design and setting

We used an exploratory qualitative design. The exploratory design was appropriate because little is known about barriers to treatment and care for PWHB in Ghana [29]. Also, the exploratory design assisted us in understanding and describing the phenomenon [30]. The study was conducted in Ghana, in a tertiary hospital in the Greater Accra region (population: approximately 4,010,050) and in a regional hospital in the Upper East region (population: approximately 1,046,545) [31]. Patients with liver conditions, including Hepatitis B, are attended to by either primary care physicians or physician specialists in the regional and tertiary hospitals. Although Ghana has a resilient national health insurance scheme (NHIS), Hepatitis B treatment and laboratory investigations including viral load are not covered under the NHIS. Typically, the average cost of Hepatitis B treatment per annum for Tenofovir medication is about Ghc 3600 ($ 670) (personal communication with a physician specialist) compared to an average annual income of about Ghc 9,600 ($1,778).

This study is part of a larger study on Hepatitis B in Ghana (see Adjei, Stutterheim, Naab, & Ruiter [32]). Ethical approval was issued by the Korle-Bu Institutional Review Board (Protocol No. KBTH-IRB 00092/2016). Informed consent (written) was obtained from the participants. In addition, information on voluntary participation and the right to withdraw were shared with the participants. Permission was also sought from the management of the data collection sites. COREQ criteria for reporting qualitative research guided the reporting of this study [33].

### Participants and recruitment

In total, 18 PWHB and 47 HCPs participated in the study. A purposive sampling technique was employed [34, 35]. PWHB were selected if they were (1) 18 years and above, (2) had tested Hepatitis B surface antigen (HBsAg) positive more than six months prior to recruitment, and (3) consented to participate. PWHB who were critically ill and had insufficient energy to participate in an interview were excluded. We therefore excluded only one patient who was found to be experiencing breathing difficulties. Also, HCPs were included if they had cared for Hepatitis B clients before but excluded if they had less than three months working experience in a department where services are provided for PWHB.

Posters with details of the study, as well as the procedure for registration were advertised in the selected health facilities. Also, some nurses assisted with the recruitment of the PWHB and the HCPs in the hospitals. Forty-nine (49) participants were recruited through nurses in the facilities and 16 participants through the advertisement. Two PWHB refused to participate in the study. One of the participants cited time constraints and the other person declined to give a reason. Another 5 HCPs did not honour the invitation because they had other programmes which coincided with the interview schedule.

### Data collection

Data were collected between February and November 2017. Participants were contacted two days before the interview through a telephone call to remind them of the time and venue for the interview/FGD. PWHB took part in face-to-face interviews whereas the HCPs participated in either interview or a focus group discussion (FGD). The use of FGD in addition to the interviews for the HCPs was to understand and describe the phenomenon comprehensively and to further ensure trustworthiness [36]. However, because of the sensitive nature of the topic and the possibility of PWHB’s responses to the study questions being quite personal, PWHB were not recruited for FGD [37].

The interviews were conducted mostly at the homes of PWHB, mainly under trees. The interviews/FGD for the HCPs were done at the nurses’ stations and the physician’s consulting rooms. Participants were briefed on the study aim, the voluntary nature of the study and measures put in place to ensure confidentiality of their information. Informed consent was signed by the participants when they were satisfied with the explanation. Also, permission was sought from each of the participants to audio record the interview/FGD sessions. The first author (CAA), who is a PhD student with a qualitative background and fluent in English conducted the interviews. All the interviews were done in English. Field notes were taken during the interviews/FGDs. In addition, background characteristics were noted including gender, age, occupation, and religion. Probing also was employed throughout the interviews/FGD [38]. The interviews lasted between 45 minutes and 1 hour whiles the FGDs with HCPs lasted approximately 1 hour and 15 minutes. Overall, 18 PWHB and 15 HCPs took part in the interviews. In addition, 4 FGD consisting of 8 HCPs in each group were done for the HCPs. Data saturated after the interview of the 14^th^ PWHB and 12^th^ HCP. Data saturation occurs when no new information emerges during an interview [30].

### Research instrument

A semi-structured interview/FGD protocol developed based on empirical literature on chronic Hepatitis B [14] guided the interviews/FGD. Additionally, a qualitative methods expert (SS) extensively reviewed the protocol. Subsequently, two PWHB and two HCPs were used to pilot the protocol. Topics explored during the interview with the PWHB included (1) whether PWHB had made contact with a HCP in the past 12 months following their diagnosis, (2) beliefs people attach to the cause of Hepatitis B in their community including themselves, (3) the kind of care and treatment PWHB use more often in their locality, (4) what makes it difficult for PWHB to visit the hospitals for treatment and care. Topics explored in the interviews and FGD for HCPs included (1) HCPs’ experiences caring for PWHB, (2) beliefs people attach to the cause of Hepatitis B, (3) the type of treatment mostly used by people with chronic Hepatitis B, (4) challenges HCPs face in managing PWHB. The interview/FGD protocols for the PWHB and the HCPs are shown in S1 Table and S2 Table respectively.

### Data analyses

We used QSR Nvivo version 10.0 to process the data. Analysis was done using the procedure of inductive thematic analysis [39]. The first author (CAA) played and listened to the audio-recordings and transcribed verbatim. Two of the authors (CAA and SS) coded the first transcript subsequently followed by discussions on the individual codes and themes generated [39].

### Rigor

We checked the preliminary findings with two representatives of the study population to establish if the findings were congruent with their views and experiences. The interview guide was piloted to establish whether the guide could elicit information that answers the research questions. In addition, transferability was ensured by providing a thick description of the study settings. Field notes and record keepings were also done to ensure confirmability [40]. Dependability was ensured by describing the research process in detail, keeping field notes as well as voice records.

## Results

### Participant characteristics

Overall, 18 PWHB participated in this study. PWHB ages ranged from 21 to 57 years (M = 35.33, SD = 8.84) and known to be positive for HBV between 1 and 7 years (M = 4.056, SD = 2.26). The participants had been diagnosed through one of the following ways: self-initiated (n = 6), physician initiated (n = 1), during outreach screening services (n = 5), and as a result of hospital protocol for pregnant women (n = 6). In terms of occupation: nurse (n = 1), unemployed (n = 4), traders (n = 4), teachers (n = 3), caterer (n = 1), student (n = 1), banker (n = 1), accountant (n = 1), sale’s manager (n = 1), and housewife (n = 1).

The HCPs comprised physicians (n = 8), nurses (n = 34), and midwives (n = 5) between 23 and 49 years of age (M = 32.77, SD = 6.91). The HCPs had worked in the field of medicine, nursing, or midwifery between 1 and 20 years (M = 6.70, SD = 4.30). Three themes and eight sub-themes emerged from the data (Summary of themes and sub-themes presented as S3 Table).

### Barriers to engagement in care

Several factors were identified as barriers to treatment and care for PWHB. These include cultural beliefs, individual level barriers, and health system-related barriers.

### Cultural beliefs as barriers

Hepatitis B is identified with various names in Ghana including “AIDS child”, Popaal” or “Kusa-yuur” (meaning pot) and “Siba” (meaning jaundice). One important set of barriers to care and treatment found in this study are cultural beliefs regarding the aetiology of Hepatitis B. These beliefs include (1) the belief that chronic Hepatitis B is a form of punishment from the gods, (2) the belief that chronic Hepatitis B is an act of witchcraft, and (3) the belief that chronic Hepatitis B is an outcome of spiritual poisoning. These cultural beliefs were found to be deeply engrained in rural communities in Northern Ghana and are important determinants of the type of treatment PWHB sought.

### Hepatitis B as punishment from the gods

First, a number of the participants including PWHB and HCPs believed that chronic Hepatitis B is a form of punishment for people who touch corpses outside their family house without permission from the landlords. In a typical rural community in Northern Ghana, selected individuals are fortified to bury corpses, popularly known as “undertakers”. These are people who have received spiritual bath and concoctions which empower them to handle and bury dead bodies. It is therefore presumed, in these areas, that PWHB might have touched a corpse without having been initiated traditionally into the society of “undertakers” and therefore were punished by the gods. One person with Hepatitis B said:

“*In my village we believe that only people who are spiritually strong must bury dead bodies because some of the ghosts can be troublesome*. *It is a common belief that people who get Hepatitis B disrespected the tradition and are punished by the gods*.*”* (PWHB, North-IDI 8)

One HCP working in one of these communities also shared his thoughts:

“*If you have Hepatitis B*, *they relate it to you handling corpses somewhere or in the family when you don’t have the powers of an undertaker*. *It requires rituals to reverse the effect*.*”* (HCP, North-IDI 3)

People who associate the causation of chronic Hepatitis B to this belief tended to seek solution from the gods through traditional priests rather than formal care.

“*Most people who believe that it is a punishment by the gods or a curse*, *once they are tested positive*, *they don’t go back to the hospital*. *They consult the gods to see what they* [the gods] *want so that they get it for them to appease them for their healing*.*”* (HCP, North-FGD 30)

“*We have a traditional priest in my village who has powerful herbs that is able to kill the Hepatitis virus from the body*. *People travel from far places to our village to get some*. *Yes*, *I know him*, *but I haven’t gone to see him for treatment before*.*”* (PWHB, North -IDI 16)

An account by a number of the HCPs revealed that they have experienced relatives of PWHB request that their family members be discharged against medical advice. This was to enable the families to perform the traditional rituals which are considered to be an effective antidote against the perceived curses. One HCP shared his experience.

“*Someone was admitted with Hepatitis B and we were treating him*. *But the family members came and insisted that we discharge him*. *They said the person has been cursed and they must go and reverse the curse before they bring him back*. *We told them that he needed to be treated first*, *but they insisted*, *and brought a letter to that effect and we discharged the man for them to go and do their rituals*.*”* (HCP, South-IDI 11)

As part of the rituals, the individual with chronic Hepatitis B is given a concoction, not for treatment, as death is perceived to be inevitable, but rather as a traditional protocol that must be observed in order to protect family members from the negative effects of the curse. It is believed that, in the absence of this traditional protocol, once a person with chronic Hepatitis B has passed away, immediate family members cannot be in proximity to the corpse of the person with chronic Hepatitis B as they will then be cursed.

A participant narrated,

“*…*.*There is a special enema or concoction that they do*. *When they do it*, *immediately for the first three days*, *the person begins to run serious diarrhoea*, *and the limbs and the pot belly will all go down*. *But they do it with the faith that the client must die*. *Because if they don’t do it and the client dies*, *the husband and his family will not be allowed to attend to the corpse*.*”* (HCP, North-FGD 4)

Further, one participant with Hepatitis B reported how traditionally dead bodies of PWHB are buried.

“*What I have seen over the years is that people who die of Hepatitis B after developing a big stomach are buried in a valley far away from town*. *We do this because such persons are seen to be cursed and so they must be far away to prevent others from getting the effect of the curse*.*”* (PWHB, North-IDI 15)

### Hepatitis B as an act of witchcraft

The second belief that acted as barrier to care and treatment was the belief that chronic Hepatitis B is caused by witches. This was often discussed in connection with people who demonstrated visible signs of chronic Hepatitis B related complications such as oedema of the feet and distended abdomen.

“*Some people think that if someone feels offended by your behaviour*, *that person can bewitch you and put certain things in your abdomen to bloat*.*”* (PWHB, North-IDI 8)

Individuals who relate the cause of chronic Hepatitis B to the act of witches tended to visit pastors for deliverance. According to the participants, in most cases, the individual condition deteriorated after visiting various prayer camps and the PWHB ended up at the hospital or died at home.

“*My father was the first person who died of Hepatitis B in my family before my brother*. *Before my father died*, *we took him to a prayer camp for many days and when he came back*, *the disease has destroyed his body*. *The stomach has become big and lost weight*. *He died few days when we sent him to the clinic just here*.*”* (PWHB, North-IDI 7)

### Hepatitis B as spiritual poisoning

The third barrier to treatment and care rooted in cultural beliefs was that Hepatitis B can be caused by spiritual poisoning. This was considered to be most common among affluent people in Ghanaian society.

“*Some people believe that if you have Hepatitis B*, *then you have been spiritually poisoned*, *either through food or through drink especially if you are rich*. *It is assumed that your enemy poisoned you without your knowledge*. *Sometimes it might be the class that you belong to or what you possess in society or the kind of job you do or it may be out of rivalry that someone somewhere decided to poison you and give you that disease*.*”* (HCP, North-IDI 6)

According to a number of the participants, concoctions prepared by herbalists are considered to be more effective in treating such poisons.

“*…*.*Some of them their perception is that it is poisoning so they will rather go and prepare their concoction and come and give it to the patient*. *Sometimes*, *they bring it to them here in the hospital*.*”* (HCP, North—FGD 19)

### Individual level barriers

Three main individual level factors were identified as barriers to treatment and care for PWHB; 1) absence of chronic Hepatitis B signs and symptoms, 2) perceived efficacy of traditional herbal medicine, and 3) PWHB’s perception that the formal care does not meet their expectations.

### Absence of chronic Hepatitis B signs and symptoms

The lack of visible signs of chronic Hepatitis B was reported to influence PWHB’s health seeking behaviour. A number of the PWHB related the lack of clinical signs and symptoms to mean that their condition was not severe or in need of monitoring by a physician.

“*I am taking nothing*. *I haven’t taken any medicine since I got to know that I have the virus*. *I don't feel sick in my body*, *not even headache*, *so why should I take drugs*? *I suspect the type of virus I have are not the dangerous type that can destroy my liver*, *so I am ok*.*”* (PWHB, South- IDI 11)

Another participant said,

“*I haven’t reported to the hospital for any treatment because I look well physically*, *and I have no complaints*.*”* (PWHB, North-IDI 14)

Evidently, the widespread myth that chronic Hepatitis B cannot, and should not, be managed appeared to play a significant role in the choice of treatment. Further, the decision of some of the participants with Hepatitis B not to engage in care was thought to be influenced by information received from HCPs. PWHB indicated being incorrectly informed about HBV management as described here below.

“*I have never been to the hospital again because the nurse said I just have to take care of my food*. *She also said if anyone says there is medicine for Hepatitis B it is a lie*, *I should not believe it*.*”* (PWHB, South-IDI 2)

### Perceived efficacy of traditional herbal medicine

In addition to the absence of symptoms acting as a barrier to Hepatitis B treatment and care, formal Hepatitis B treatment and care was reported to also be inhibited by the perceived efficacy of traditional herbal medicine. It was evident in this study that herbal medicines were used by PWHB more often than formal care with many participants indicating that herbal medicines are an effective treatment option for chronic Hepatitis B. Some participants linked the high utilisation of herbal medicine to extensive advertisements of herbal products on various mass media platforms in Ghana.

“*Most people think that hospital medication cannot cure the disease*. *I know of someone who went to the hospital and she was diagnosed Hepatitis B positive*. *After that*, *she said the hospital treatment is not able to cure Hepatitis B so they came home for herbal treatment*.*”* [PWHB—South]

According to a number of the participants, formal healthcare facilities are often used as last resort by PWHB, only after herbal medicine fails to achieve the expected effect.

“*Sometimes when they come to the hospital*, *they later admit that they took some herbal preparation*, *but it did not work and that’s why they want to try the hospital drugs*.*”* (PWHB, South-IDI 3)

According to one physician specialist, herbal medicine is highly problematic because often PWHB who use herbal medicines report to the hospital with extensive liver damage which makes the condition difficult to manage.

“*We have a lot of people coming in with complications*. *They don’t come in with just Hepatitis B*. *The problem comes from the herbal medications and concoctions*.*”* [Physician specialist–South, IDI 15]

### Formal care does not meet expectations

A third individual level barrier found was that formal care does not meet PWHB’s expectations. A number of the PWHB reported that they feel that some health facilities are unable to meet their health needs. Specifically, there were disappointed because they expected physicians to prescribe drugs for them on their first visit. Those whose expectations were perceived to be unmet tended to lose confidence in the hospital-based management and thus often opted for alternative treatment, usually herbal medicine. One person with chronic Hepatitis B said,

“*When I come*, *they won’t give me medicine*, *they won’t give me injection*. *They keep saying that I have to be monitored to see whether the virus will start reacting*. *Is just like something is lying down*. *You just see that this is a scorpion and is lying down but it is not biting you*. *Why can’t it be killed since it is known to be dangerous*. *That is what they are doing in my case*. *They are saying I should come to be monitored to see when it will start reacting before they arrest it*.*”* (PWHB, South-IDI 18)

Another participant also expressed her expectation.

“*I want them to give me something like medicine or injection that will kill the viruses completely*.*”* (PWHB, South- IDI 1)

Participants who lacked an understanding of the processes involved in the management of HBV felt that their physicians lacked the expertise needed to effectively manage their condition.

“*I think the doctors don’t know what to do for me*. *When I come they tell me they are still observing me*. *The story is still the same*, *even today*.*”* (PWHB, North-IDI 16)

### Health-system related barriers

In addition to individual level barriers, we also identified health-system factors that acted as barriers to care and treatment for PWHB. These factors included the high cost of hospital-based care, and inadequate Hepatitis B education for patients from HCPs.

### High cost of hospital-based care

The high cost of chronic Hepatitis B management was widely reported by PWHB as an important barrier to treatment and care. The cost of the services spanned from laboratory investigations to prescription drugs. A participant with chronic Hepatitis B recounted her challenge,

“*One major problem is money because the labs involve a lot*. *The recent one I just did is about Ghc582* [$112]. *It sometimes prevents you from coming for the review*. *I am wondering the fee for those on treatment*.*”* (PWHB, South- IDI 17)

In addition, concerns about lost income due to appointments were reported. This was particularly the case among participants who had to miss work in order to come to a physician’s appointment.

“*Since 2014 up to date*, *nothing has been done for me*. *I have been doing tests only*. *Since I came here it has been the same thing*. *Only that I am spending money*. *I am supposed to be at work but I have to stop and be here*.*”* (PWHB, South- IDI 5)

The challenge posed by the high costs for the management of chronic Hepatitis B reported by PWHB was also confirmed by a physician specialist.

“*For those who come in with Hepatitis B*, *if they can be treated the issue is cost*. *The drug is quite expensive for a number of patients*.*”* (Physician Specialist, South-IDI 15)

Unlike other conditions such as hypertension, malaria, and HIV, where treatment is provided free of charge in Ghana, Hepatitis B services have fees. Some participants expressed concern about the exclusion of Hepatitis B drugs from the national health insurance scheme and requested for its inclusion.

“*What I would advise is if the health insurance can do something so that at least people with such conditions can enjoy their medications being covered by the health insurance*.*”* (HCP, North-FGD 21)

### Inadequate Hepatitis B education for patients from HCPs

In addition to the high cost of hospital-based care reported above, a number of the participants said they did not know they needed monitoring and follow up. One participant said,

“*I haven’t received any form of support from the hospital*. *Before I gave birth*, *I asked the nurse if I could take medicine but she said nothing except healthy food*.*”* (PWHB, North-IDI 7)

According to a physician participating in this study, no formalised education programme on Hepatitis B is done by HCPs in the area. There are, however, a few individuals who do public campaigns about HBV.

“*There is no real form of education to make the general public aware what Hepatitis B really is*. *To the best of my knowledge*, *there are only a few sporadic nurses who once in a while mention about Hepatitis B*. *Aside that*, *people do not really have any form of education about Hepatitis B or any platform where they can be taught about Hepatitis B*.*”* (HCP, South-IDI 4)

HCP participants further contended that the existing lack of knowledge gap about Hepatitis B is often filled by the traditional medicine practitioners who appear to fuel inaccurate and dangerous information about the HBV. One HCP recounted her observation,

“*…*.*We are making the herbal people misinform a lot of people*. *Sometimes you watch television or advert and it is funny*. *All that these herbalists say is*, *come and in three days*, *your Hepatitis B will be gone*. *Come*, *we have cure within two hours*.*”* (HCP, South-IDI 10)

In the absence of clear information from HCP, PWHB’s conceptions of Hepatitis B, its course, and its treatment are formed by information from herbal medicine practitioners.

## Discussion

This study explored barriers to treatment and care for PWHB in Ghana. Three main cultural beliefs regarding the aetiology of Hepatitis B that act as barriers to care and treatment were identified. Furthermore, individual level barriers and health system-related barriers were found.

Consistent with other studies in Ghana and elsewhere [23, 4–44], this study identified misconceptions regarding the aetiology of Hepatitis B as reflected in our participants’ beliefs. For example, a study that explored the perceptions and understanding of HBV in the Upper West Region of Ghana found that people linked the signs and symptoms of chronic Hepatitis B to witchcraft and poison [23]. Also, in a study with pregnant women in peri-urban Ghana, curses were perceived to be a possible cause of Hepatitis B [24]. These findings are not astounding because, in most Ghanaian societies, diseases with unusual presentations are attributed to evil influences [45–46]. We also identified that the belief PWHB attach to the aetiology of HBV seems to have an influence on the type of treatment they seek. Those who believe that chronic Hepatitis B is a punishment by the gods tend to visit the traditional priests for treatment whereas those who link HBV to the acts of witches and/or spiritual poisoning often consult pastors for deliverance and herbalists for treatment, respectively. This is worrying as these beliefs can delay health seeking, lead to poor health outcomes, and contribute to possible risk of HBV transmission to household relations [24, 47].

We also found that the absence of visible signs and symptoms of chronic Hepatitis B is a barrier to care and treatment. Some of the participants misinterpreted the lack of clinical manifestations of chronic Hepatitis B to mean that HBV does not need treatment. This is not surprising given that Wu and colleagues [48], in their study of socio-cultural factors that potentially affect the institution of prevention and treatment strategies for Hepatitis B in Chinese Canadians, found that a number of the participants were unaware that Hepatitis B progression to the stage of complication could occur in the absence of symptoms. The reality is that many people with HBV do not experience early clinical symptoms [1]. This particular characteristic of the infection appears to be one of the reasons why people delay care following HBV diagnosis and thus needs to be clearly conveyed to PWHB.

Perceived efficacy of traditional herbal medicine was also reported to be an important barrier to treatment and care. Herbal medicines are often preferred by Africans more broadly because they are easily accessible, less expensive, perceived as effective, and convenient [47, 49]. In Ghana, a number of companies advertise herbal products in both electronic and print media including television and radio [50], and herbal medicines seem to be the first line treatment for several diseases including viral Hepatitis B. These indigenous medicines are often perceived to be more effective than conventional medicines [50]. However, the proclaimed effectiveness of these medicines is not based on clinical trials but self-reported by consumers [50]. Unfortunately, herbal medicine use is problematic because it delays formal treatment and a number of people conceal their use of herbal medicine from HCPs [47, 49] unless they experience side effects. In fact, a recent study conducted in Ghana found that 86% of herbal medicine users failed to disclose their previous history of herbal product use to HCPs [49]. In our findings, a physician specialist spoke of PWHB who developed liver complications after using herbal products. Similar studies conducted in other countries have also documented the role herbal medicines can play in the management of viral Hepatitis B [14, 17, 47]. For example, in Burkina Faso, Giles-Vernick et al. [14] reported a high demand for herbal medicine for chronic Hepatitis B treatment and outlined that some HCPs even encouraged clients with chronic Hepatitis B to consult herbal practitioners for treatment instead of physicians in the formal health sector. In Singapore, herbal medicines are used as well for the treatment of chronic Hepatitis B but once found ineffective, their use tends to be discontinued [14].

Another finding of this present study was that some PWHB felt that the hospital-based care does not meet their expectations. Their expectations were to receive prescription drugs and injections that eliminate the HBV on their first visit without necessarily going through periodic clinical monitoring. However, the AASLD practice guideline outlines criteria for PWHB eligible for treatment and this is not always in line with PWHB’s expectations [4]. Only PWHB with elevated viral load, elevated serumalanine aminotransferase (ALT) levels, and evidence of liver fibrosis are considered candidates for treatment [4]. Given these unmet expectations, it is possible that HCPs do not sufficiently discuss the relevance of clinical monitoring with their Hepatitis B patients and that this, in turn, contributes to the perceptions of PWHB’s that their health care needs are unmet.

In additon to participant’s perceptions that care does not meet their expectations, the high costs of chronic Hepatitis B treatment in the formal health system was noted as an important barrier to care and treatment. Although no study estimating the cost of treatment and care of PWHB in Ghana has been conducted, a number of our study participants indicated that the management of HBV comes at a high economic cost to the individual and the family. The situation is further compounded when the PWHB is not a suscriber of the National Health Insurance Scheme (NHIS). Other related studies have also raised concerns about the high costs of Hepatitis B management in other locales, including the United States, Iran, and other poor resource settings [5, 16, 51].

Finally, this study found that education about Hepatitis B for PWHB from HCPs was lacking. The participants with chronic Hepatitis B revealed that they were often unsure about what to do, and where to go, after diagnosis. This is not surprising in the study setting considering that a previous study identified inadequacies in Hepatitis B knowledge among some HCPs in Ghana [52]. There is thus a need for capacity training among HCPs so that they better understand, and convey to their patients, the complexities of chronic Hepatitis B including the management protocol [16, 53–54].

Our findings have some theoretical and practical implications. In terms of theory, this study is one of the few studies providing a Sub-Saharan African perspective on the role of cultural beliefs in care engagement for PWHB. In terms of practice, there are two main implications. First, given that the high costs of hospital based care was considered an important barrier to engagement in care for PWHB, we recommend including the required Hepatitis B laboratory investigations such as viral load, and the recommended treatment, in the NHIS. Second, in light of our finding that cultural beliefs about aetiology impede care engagement and given the individual level barriers established, we recommend increasing health care providers Hepatitis B knowledge and capacity to, in a culturally sensitive fashion [55], discuss with patients (1) myths about aetiology and the lack of efficacy of traditional herbal medicines, and (2) patients’ expectations of care and the need to monitor even in the absence of symptoms.We further recommend, the Ghana Food and Drugs Board Authority strengthens their regulatory mechanism to ensure the safety of the consumers of herbal products purported to be effective for the treatment of chronic Hepatitis B.

This study had both strengths and limitations. One of the methological strengths of our study is the recruitment of both PWHB and HCPs, which is reflective of data source triangulation and contributes to rigour. A possible limitation in this study is the possibility of recall bias because participants had to retrospectively recount their experiences. However, this was minimised by asking follow up questions. In terms of future research, we recommend following this study with a survey study that measures the frequency and relative importance of these barriers among PWHB in Ghana. This is partuclarly important given that a small number of participants took part in this study limiting the generalisability of the findings. Also, evaluating the effectiveness of herbal medicine for HBV treatment is worth considering in the study area.

## Conclusion

This study has explored and outlined barriers to treatment and care for PWHB in Ghana, paying particular attention to beliefs about aetiology that can act as a barrier to care for PWHB. We found that cultural beliefs about aetiology of HBV in addition to individual and health system-related factors act as barriers to treatment and care for PWHB in Ghana. We therefore recommend increasing knowledge of PWHB and HCPs in a culturally sensitive fashion to correct the myths about aetiology of Hepatitis B. This we believe will improve the health seeking behaviour of PWHB. Given that high cost of hospital based care was considered an important barrier to engagement in care for PWHB, we recommend including the required Hepatitis B laboratory investigations such as viral load, and the recommended treatment in the NHIS.

## Supporting information

S1 Table Interview protocol for people with hepatitis B. (DOCX)

S2 Table Interview/FGD protocol for healthcare providers. (DOCX)

S3 Table Summary of findings. (DOCX)

## Abbreviations

HBV: Hepatitis B Virus
PWHB: People with Hepatitis B
HCPs: Healthcare Providers
