# Supplementary material for: Barriers to chronic Hepatitis B treatment and care in Ghana: A qualitative study with people with Hepatitis B and healthcare providers
Source: PLoS One. 2019 Dec 3;14(12):e0225830. doi: 10.1371/journal.pone.0225830 (PMC6890212; doi:10.1371/journal.pone.0225830)
Supplement: S1 Table — (DOCX) [file pone.0225830.s001.docx]

**S1 Table. Interview protocol for people with hepatitis B**

| **Background information**   - Age - Gender - Marital status - Occupation - Year first diagnosed with hepatitis B? - How participant got tested (self-request, general screening exercise, recommendation by physician, employment requirement, pre-marital requirement etc.)   **Barriers to care and treatment**   - Have you made any contact with a health care provider (physician) in the past 12 months following your diagnosis? Probe - Can you share with me beliefs people attach to the cause of hepatitis B in your community? Probe - Can you share with me how you seek for care and treatment in your locality? Probe - What are your health needs and are they met when you visit the hospital? Probe - What makes it difficult for you to visit the hospitals for care and treatment? Probe - Is the health care system supportive? Are the healthcare providers supportive? Probe - How are you treating the disease? Probe |
| --- |
