# Supplementary material for: Barriers to chronic Hepatitis B treatment and care in Ghana: A qualitative study with people with Hepatitis B and healthcare providers
Source: PLoS One. 2019 Dec 3;14(12):e0225830. doi: 10.1371/journal.pone.0225830 (PMC6890212; doi:10.1371/journal.pone.0225830)
Supplement: S2 Table — (DOCX) [file pone.0225830.s002.docx]

**S2 Table. Interview/FGD protocol for healthcare providers**

| **Background Information**   - Age - Gender - Marital status - Occupation   **Barriers to care and treatment**   - Have you attended to/nursed someone infected with Hepatitis B before? - If yes, can you share with me your experience? Probe - Can you share with me the beliefs people attach to the causes of Hepatitis B? Probe - What treatment options do people with Hepatitis B mostly explore and why? Probe - What challenges do you face when managing these patients? Probe |
| --- |
