# Supplementary material for: Barriers to chronic Hepatitis B treatment and care in Ghana: A qualitative study with people with Hepatitis B and healthcare providers
Source: PLoS One. 2019 Dec 3;14(12):e0225830. doi: 10.1371/journal.pone.0225830 (PMC6890212; doi:10.1371/journal.pone.0225830)
Supplement: S3 Table — (DOCX) [file pone.0225830.s003.docx]

**S3 Table. Summary of findings**

| **Themes** | **Sub-Themes** |
| --- | --- |
| 1. Cultural Beliefs | - The belief that chronic Hepatitis B is a punishment from the gods - The belief that bewitchment contributes to chronic Hepatitis B - The belief that chronic Hepatitis B is caused by spiritual poison |
| 1. Individual level barriers | - Absence of chronic Hepatitis B signs and symptoms - Perceived efficacy of traditional herbal medicine - Formal care does not meet the expectations of PWHB |
| 1. Health system-related barriers | - High cost of hospital-based care - Inadequate Hepatitis B education for patients from HCPs |
